# Polypharmacy, Potentially Inappropriate Medications, and Drug-Drug Interactions in Vulnerable Older Adults With Advanced Cancer Initiating Cancer Treatment

**DOI:** 10.1093/oncolo/oyac053

**Published:** 2022-03-28

**Authors:** Erika Ramsdale, Mostafa Mohamed, Veronica Yu, Ethan Otto, Katherine Juba, Hala Awad, Kiran Moorthi, Sandy Plumb, Amita Patil, Nicholas Vogelzang, Elie Dib, Supriya Mohile

**Affiliations:** James P. Wilmot Cancer Center, University of Rochester Medical Center, Rochester, NY, USA; James P. Wilmot Cancer Center, University of Rochester Medical Center, Rochester, NY, USA; James P. Wilmot Cancer Center, University of Rochester Medical Center, Rochester, NY, USA; James P. Wilmot Cancer Center, University of Rochester Medical Center, Rochester, NY, USA; Department of Pharmacy Practice, Wegmans School of Pharmacy, Rochester, NY, USA; Department of Pharmacy, University of Rochester Medical Center, Rochester, NY, USA; Clinical & Translational Science Institute, University of Rochester Medical Center, Rochester, NY, USA; James P. Wilmot Cancer Center, University of Rochester Medical Center, Rochester, NY, USA; James P. Wilmot Cancer Center, University of Rochester Medical Center, Rochester, NY, USA; School of Nursing, Johns Hopkins University, Baltimore, MD, USA; Nevada Cancer Research Foundation, NCI Community Oncology Research Program, Las Vegas, NV, USA; St. Joseph Mercy Cancer Center, Ypsilanti, MI, USA; James P. Wilmot Cancer Center, University of Rochester Medical Center, Rochester, NY, USA

**Keywords:** polypharmacy, medication use, drug-drug interactions, geriatric oncology, potentially inappropriate medications, supportive care

## Abstract

**Purpose:**

Polypharmacy is prevalent in older adults starting cancer treatment and associated with potentially inappropriate medications (PIM), potential drug-drug interactions (DDI), and drug-cancer treatment interactions (DCI). For a large cohort of vulnerable older adults with advanced cancer starting treatment, we describe patterns of prescription and nonprescription medication usage, the prevalence of PIM, and the prevalence, severity, and type of DDI/DCI.

**Methods:**

This secondary analysis used baseline data from a randomized study enrolling patients aged ≥70 years with advanced cancer starting a new systemic cancer treatment (University of Rochester Cancer Center [URCC] 13059; PI: Mohile). PIM were categorized using 2019 Beers criteria and Screening Tool of Older Persons’ Prescriptions. Potential DDI/DCI were evaluated using Lexi-Interact Online. Medication classification followed the World Health Organization Anatomical Therapeutic Chemical system. Bivariate associations were evaluated between sociodemographic and geriatric assessment (GA) measures and medication measures. Chord diagrams and network analysis were used to understand and describe DDI/DCI.

**Results:**

Among 718 patients (mean age 77.6 years), polypharmacy (≥5 medications), excessive polypharmacy (≥10 medications), and ≥1 PIM were identified in 61.3%,14.5%, and 67.1%, respectively. Cardiovascular medications were the most prevalent (47%), and nonprescription medications accounted for 26% of total medications and 40% of PIM. One-quarter of patients had ≥1 potential major DDI not involving cancer treatment, and 5.4% had ≥1 potential major DCI. Each additional medication increased the odds of a potential major DDI and DCI by 39% and 12%, respectively. Polypharmacy and PIM are associated with multiple GA domains.

**Conclusion:**

In a cohort of vulnerable older adults with advanced cancer starting treatment, polypharmacy, PIM, and potential DDI/DCI are very common. Nonprescription medications are frequently PIMs and/or involved in potential DDI/DCI.

## Teaser

Polypharmacy, the concurrent usage of multiple medications, is common in older adults with cancer and is associated with adverse outcomes. This article characterizes polypharmacy, potentially inappropriate medications, and potential drug-drug interactions and drug-cancer treatment interactions in a large cohort of older adults with advanced cancer.

**Implications for Practice**

Polypharmacy is prevalent in older adults with cancer. It increases the risk of potentially inappropriate medications (PIMs), potential drug-drug interactions (DDI), and potential drug-cancer therapy interactions (DCI). This study describes medication usage in a large cohort of older patients with cancer starting chemotherapy in community oncology practices, including a detailed examination and visualization of the medications most often contributing to potential DDI/DCI. Notably, nonprescription medications, not often included in other analyses, are commonly PIMs and/or contribute to potential DDI/DCI. This study yields highly pragmatic information to help focus awareness and attention on the medications which may pose risk in older patients starting cancer treatment.

## Introduction

Polypharmacy, the concurrent usage of multiple medications, is common in older adults with cancer^1^ and associated with numerous adverse outcomes. Older adults are more likely than their younger counterparts to be prescribed multiple medications due to age-related multimorbidity,^2^ frailty, and other geriatric syndromes.^3,4^ Fragmented care across multiple specialties^5^ and “prescribing cascades,”^6^ the prescription of additional medications to mitigate adverse effects of another medication, are also common. Polypharmacy engenders a higher risk of patients taking “potentially inappropriate medications” (PIMs),^7^ drugs which have risks higher than anticipated benefits. Polypharmacy and PIMs are associated with functional decline,^8^ falls,^9^ hospitalizations,^10^ and mortality^8,11^ in older adults.

Older adults with cancer, who are more likely to have frailty, disability, and geriatric syndromes than older patients without cancer,^12^ may be at particularly high risk of adverse events from PIMs. PIMs have been shown to decrease tolerance of cancer treatment^13^ and worsen outcomes including physical function.^14,15^ Polypharmacy and PIMs also increase the risk of clinically significant drug-drug interactions (DDI) and drug-cancer treatment interactions (DCI) in those receiving cancer treatments.^16^

Polypharmacy and PIMs are understudied in older adults with cancer, and available data are heterogeneous. Estimates of polypharmacy prevalence vary widely due to inconsistent definitions.^17^ Although the most common definition for polypharmacy in the literature is ≥5 medications,^18^ cut-off values range from 3 to 10 medications.^14^ The use of ≥10 medications is often called “hyperpolypharmacy” or “excessive polypharmacy.”^2^ Moreover, studies often include only scheduled prescription medications, overlooking over-the-counter (OTC) medications, complementary/alternative medications,^2^ and medications taken on an as-needed basis. These omitted categories include many common PIMs in older adults (eg, nonsteroidal anti-inflammatory drugs [NSAIDs], antihistamines, and proton pump inhibitors [PPIs]),^19^ as well as medications that increase the risk of DDIs.

This study aims to characterize polypharmacy, PIMs, and potential DDI/DCI in a large cohort (*n* = 718) of vulnerable older adults with advanced cancer recruited to a national prospective cluster-randomized trial of geriatric assessment (GA), conducted in community-based (“real world”) oncology practices.^20^

## Methods

### Study Design

This is a secondary analysis of baseline data from a nationwide, multicenter, cluster-randomized study that assessed whether providing information regarding GA to community oncologists reduced clinician-rated grade 3-5 chemotherapy toxicity in older patients with advanced cancer starting a new cancer treatment regimen (Geriatric Assessment for Patients [GAP70+] study; University of Rochester Cancer Center [URCC] 13059, PI: Mohile; ClinicalTrials.gov identifier: NCT02054741).^20^ Enrollment of participants occurred between July 2014 and March 2019. A polypharmacy log, including medication name, dose, primary indication, start and end dates, frequency, and route of administration was completed for all the participants at baseline by a clinical research associate at each study site. Recorded medication names were all converted to generic names prior to analysis, and duplicate medications (ie, if a medication was accidentally recorded twice) were eliminated. The primary study was conducted by the URCC NCI Community Oncology Research Program Research Base and approved by the Institutional Review Boards at participating sites. All participants provided written informed consent.

### Participants

Eligible patients were (1) aged ≥70 years, (2) diagnosed with incurable stage III/IV solid tumor or lymphoma, (3) impaired in at least one GA domain excluding the polypharmacy domain, and (4) planning to start a new cancer treatment regimen with a high risk of grade 3-5 toxicity based on Common Terminology Criteria for Adverse Events, v4.^21^ Eligible regimens were determined based on enrolling physicians’ discretion and were reviewed by blinded clinical staff at the URCC Research Base.

### Medication Review

At baseline and prior to initiation of the new cancer treatment regimen, the polypharmacy log captured regular medications (prescription, OTC, and complementary/alternative medications) received by the patient within the prior 2 weeks. Cancer therapies and supportive care medications were collected in a separate log and not included in the total medication count, PIM evaluation, or DDI/DCI analyses. Polypharmacy was defined as using ≥5 regular medications while excessive polypharmacy was defined using ≥10 regular medications.

PIMs were categorized using 2 screening tools: the 2019 Beers criteria,^22^ endorsed by the American Geriatrics Society, and the Screening Tool of Older Person’s Prescriptions (STOPP) criteria,^23^ a European screening tool developed on the basis of expert consensus and evidence-based criteria. Drug interactions were reviewed using Lexi-Interact Online.^24^ Potential DDIs were categorized as any category C (monitor therapy), D (consider therapy modification), or X (avoid combination) interactions between 2 regular (non-cancer treatment) drugs. Potential major DDI are any category D or X interactions. Potential DCI is any category C, D, or X interactions between a regular drug and a cancer treatment drug (not including supportive care medications), and potential major DCI is any category D or X interactions between a regular drug and a cancer treatment drug.

Medication classes were identified using the 5 nested levels of the World Health Organization (WHO) Anatomical Therapeutic Chemical (ATC) classification system.^25^ In this analysis, we identified the first 3 levels of classification (first level: Anatomical or Pharmacological group; second level: Pharmacological or Therapeutic subgroup, and third level: Chemical, Pharmacological or Therapeutic subgroup). Substances not explicitly listed in the WHO ATC Index were classified by their main active ingredient and/or primary indication according to the Guidelines for ATC classification.^25^

### Baseline Variables

Socio-demographic variables included age, gender, race (White, Black, and others), education (less than high school, high school graduate, and some college or more), marital status (never married, married/domestic partner, and separated/widowed/divorced), and income (≤$50 000 and >$50 000/declined to answer). Clinical variables included cancer type (gastrointestinal, lung, others), cancer stage (stage III, stage IV), line of palliative treatment (first versus second-line or later), life expectancy estimated by the physician (≤1 year and >1 year), and physician-reported Karnofsky Performance Score (KPS, 40-60, 70-80, and 90-100).^26,27^ GA domains were captured using validated tools with established cut-offs for impairment including comorbidity, functional status (measured by the ability to complete activities of daily living), physical performance (using objective measures), cognition, social support, psychological health, and nutritional status. These domains have been detailed previously (Supplementary Table 1).

### Data Analysis

Descriptive analyses were performed to evaluate medication variables related to polypharmacy, PIM, and drug interactions. Means and SD were generated for continuous data, and proportions and frequencies for categorical data. Bivariate associations between baseline variables, polypharmacy/PIM, and potential DDI/DCI were calculated by using Fisher’s exact test and Pearson’s χ^2^ test for categorical variables and independent *t*-test for continuous variable (total number of medications). Two-sided *P*-values of <.05 were considered statistically significant. Chord diagrams were used to visualize the frequency of potential DDI and DCI between medications, at all levels of WHO ATC classification. Methods of social network analysis^28^ were used to examine and quantify the characteristics and interconnectedness of DDI and DCI “networks.” Assuming a network, where each medication or medication subgroup is a “node,” and connections between nodes represented a DDI, we measured density (existing connections as a fraction of all possible connections between nodes), diameter (the maximum shortest travel distance between any 2 nodes), and triadic closure (the percentage chance that, if 2 nodes are connected to a third node, the 2 nodes are themselves connected) for the top 3 levels of the WHO ATC classification as well as the individual medication level. Analyses were conducted with SAS 9.4 and Python 3.7.4.

## Results

### Baseline Characteristics

Among 718 participants, the mean age was 77.2 years (range 70-96); 43.3% (*n* = 311) identified as female, 87.5% (*n* = 628) identified as non-Hispanic White, and 87.5% (*n* = 628) had stage IV cancer. The mean number of GA domain impairments was 4.5 (SD 1.6), 67.5% were considered impaired on the comorbidity scale (≥1 comorbidity that affected the patient a “great deal,” or ≥3 that affected the patient “somewhat” on the modified Older American Resources and Services comorbidity scale) and 57.5% (*n* = 412) were considered functionally impaired based on GA (Table 1). The most common non-cancer comorbidities were hypertension (62.0%, *n* = 445), arthritis (49.3%, *n* = 354), heart diseases (30.1%, *n* = 216), and diabetes mellitus (24.8%, *n* = 178). The average number of comorbidities per patient was 3.2 (range 0-9).

**Table 1.** Association of baseline variables and polypharmacy (≥ 5 medications).

Variable	Category	All patients	Polypharmacy	No polypharmacy	POR	95% CI	
*N*		718 (100%)	440 (61.3%)	278 (38.7%)			
Age, years^*^	70-74	271 (37.7%)	162 (36.8%)	109 (39.2%)	Ref	—	—
	75-79	225 (31.3%)	130 (29.6%)	95 (34.2%)	0.92	0.64	1.32
	≥80	222 (30.9%)	148 (33.6%)	74 (26.6%)	1.35	0.93	1.95
Gender^a^	Male	405 (56.4%)	245 (55.8%)	160 (57.8%)	Ref	—	—
	Female	311 (43.3%)	194 (44.2%)	117 (42.2%)	1.08	0.80	1.47
Race^b^	White	628 (87.5%)	384 (87.67%)	244 (88.1%)	Ref	—	—
	Black	52 (7.2%)	30 (6.85%)	22 (7.9%)	0.87	0.49	1.54
	Others	35 (4.9%)	24 (5.84%)	11 (4.0%)	1.39	0.67	2.88
Education^a^	< High school	111 (15.5%)	70 (15.95%)	41 (14.8%)	Ref	—	—
	High school	244 (34.0%)	146 (33.26%)	98 (35.38%)	0.87	0.55	1.39
	College or above	361 (50.3%)	223 (50.80%)	138 (49.82%)	0.95	0.61	1.47
Income^a^	≤$50 000	371 (51.7%)	235 (53.53%)	136 (49.10%)	Ref	—	—
	>50 000	190 (26.5%)	114 (25.97%)	76 (27.44%)	0.87	0.61	1.24
	Declined to answer	155 (21.6%)	90 (20.50%)	65 (23.47%)	0.80	0.55	1.18
Marital status^a^	Single	17 (2.4%)	9 (2.1%)	8 (2.9%)	Ref	—	—
	Married/domestic partnership	449 (62.5%)	286 (65.2%)	63 (58.8%)	1.56	0.59	4.12
	Separated/ widowed/divorced	250 (34.8%)	144 (32.8%)	106 (38.3%	1.21	0.45	3.23
Cancer type	Gastrointestinal	246 (34.2%)	143 (32.5%)	103 (37.4%)	Ref	—	—
	Genitourinary	109 (15.2%)	65 (14.8%)	44 (15.8%)	1.07	0.68	1.70
	Gynecological	43 (6.0%)	29 (6.6%)	14 (5.0%)	1.51	0.76	2.99
	Breast	56 (7.8%)	31 (7.1%)	25 (9%)	0.90	0.50	1.62
	Lung	180 (25.1%)	122 (27.7%)	58 (20.9%)	1.53	1.02	2.29
	Lymphoma	46 (6.4%)	28 (6.4%)	18 (6.5%)	1.13	0.59	2.15
	Others	38 (5.3%)	22 (5%)	15 (5.4%)	1.07	0.53	2.16
KPS	20-60	93 (13.0%)	71 (16.2%)	22 (7.9%)	Ref	—	—
	70-80	379 (52.8%)	236 (53.8%)	143 (51.6%)	**0.51**	**0.30**	**0.86**
	90-100	244 (33.9%)	132 (30.1%)	112 (40.4%)	**0.37**	**0.21**	**0.63**
Life expectancy^c^	≤12 months	238 (33.1%)	148 (34.1%)	90 (32.5%)	Ref	—	—
	>12 months	473 (66.0%)	286 (65.9%)	187 (67.5%)	0.93	0.68	1.28

Two patients had missing data.

Three had missing data.

Seven had missing data.

*P* < .05 for age as a continuous variable.

Abbreviations: CI, confidence interval; KPS, Karnofsky Performance Status; POR, prevalence odds ratio.

Bolded values are statistically significant.

### Prevalence of Polypharmacy and PIMs

The cohort reported 4176 occurrences of 517 distinct regular medications (prescription and nonprescription), with a median number of 5 medications per patient (range 0-24). Polypharmacy (≥5 medications) and excessive polypharmacy (≥10 medications) were identified in 61.3% (*n* = 440) and 14.5% (*n* = 104) of patients, respectively.

**Figure 1.**
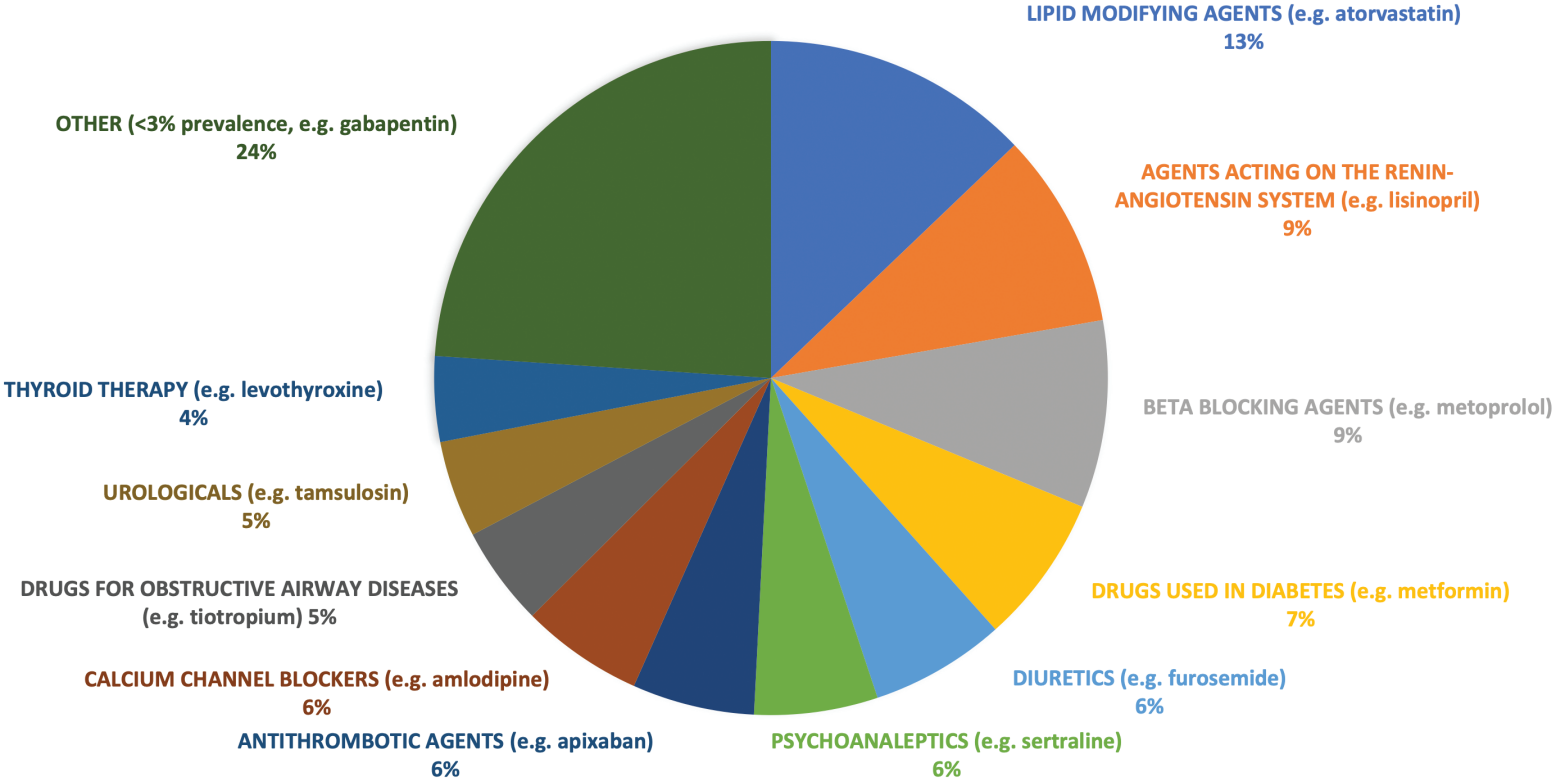
The most prescribed therapeutic subgroups (WHO ATC level 2 classification).

**Figure 2.**
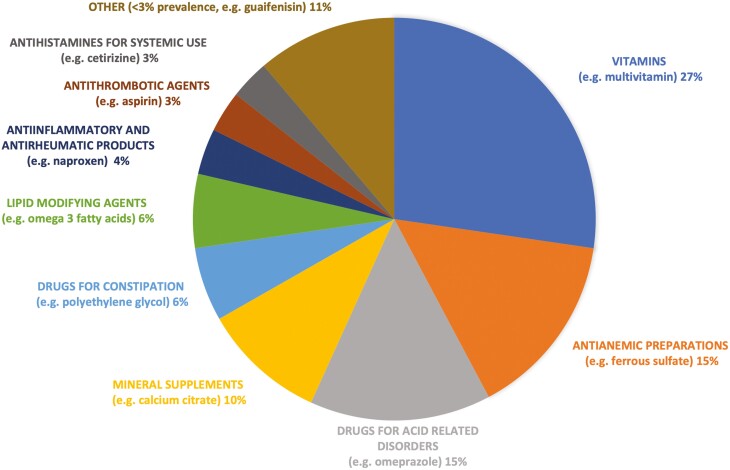
The most nonprescription therapeutic subgroups (WHO ATC level 2 classification).

Among prescribed medications (*n* = 3063 occurrences, 73.3% of total medications), cardiovascular agents were the most common (47.0%, *n* = 1440). Other commonly prescribed drug classes included nervous system agents such as antidepressants (13.0%, *n* = 397), alimentary tract, and metabolism medications such as antidiabetics (13.2%, *n* = 405), and systemic hormonal preparations (6.5%, *n* = 199). Lipid modifying agents (13.0%, *n* = 399), agents acting on renin-angiotensin system (9.5%, *n* = 291), beta blockers (9.5%, *n* = 291), drugs used in diabetes (7.2%, *n* = 222), and diuretics (6.6%, *n* = 201) were the most prescribed therapeutic subgroups (WHO ATC level 2 classification) (Fig. 1). Nonprescription medications accounted for 26.7% of all medications (*n* = 1113), with vitamins (27.0%, *n* = 301), anti-anemic preparations (14.9%, *n* = 166), drugs for acid related disorders (14.6%, *n* = 162), and mineral supplements (9.8%, *n* = 110) as the most common nonprescription therapeutic subgroups (WHO ATC level 2) reported (Fig. 2).

Based on the 2019 Beers Criteria, 447 patients (62.3%) received ≥1 PIM (range 0-8), including PPIs (22%), benzodiazepines (13%), NSAIDs (9%), and first-generation antihistamines (8%). By STOPP criteria, 206 patients (28.7%) received ≥1 PIM, including first-generation antihistamines (13%), beta-blockers (12%), benzodiazepines (9%), and NSAIDs (6%). Applying both tools, 482 patients (67.1%) received ≥1 PIM. Nonprescription medications accounted for 41.8% and 33.0% of PIM identified by Beers and STOPP criteria, respectively.

### Prevalence of DDI

There were 1854 potential DDI identified among 490 patients. Of these, 1589 were category C affecting 64.3% of patients, 280 were category D in 22.0% of patients, and 34 were category X in 4.2% of the participants. Approximately 25% (*n* = 177) of the study participants had at least one potential major DDI not involving cancer treatment, and 5.4% (*n* = 39) had at least one potential major DCI.

**Figure 3.**
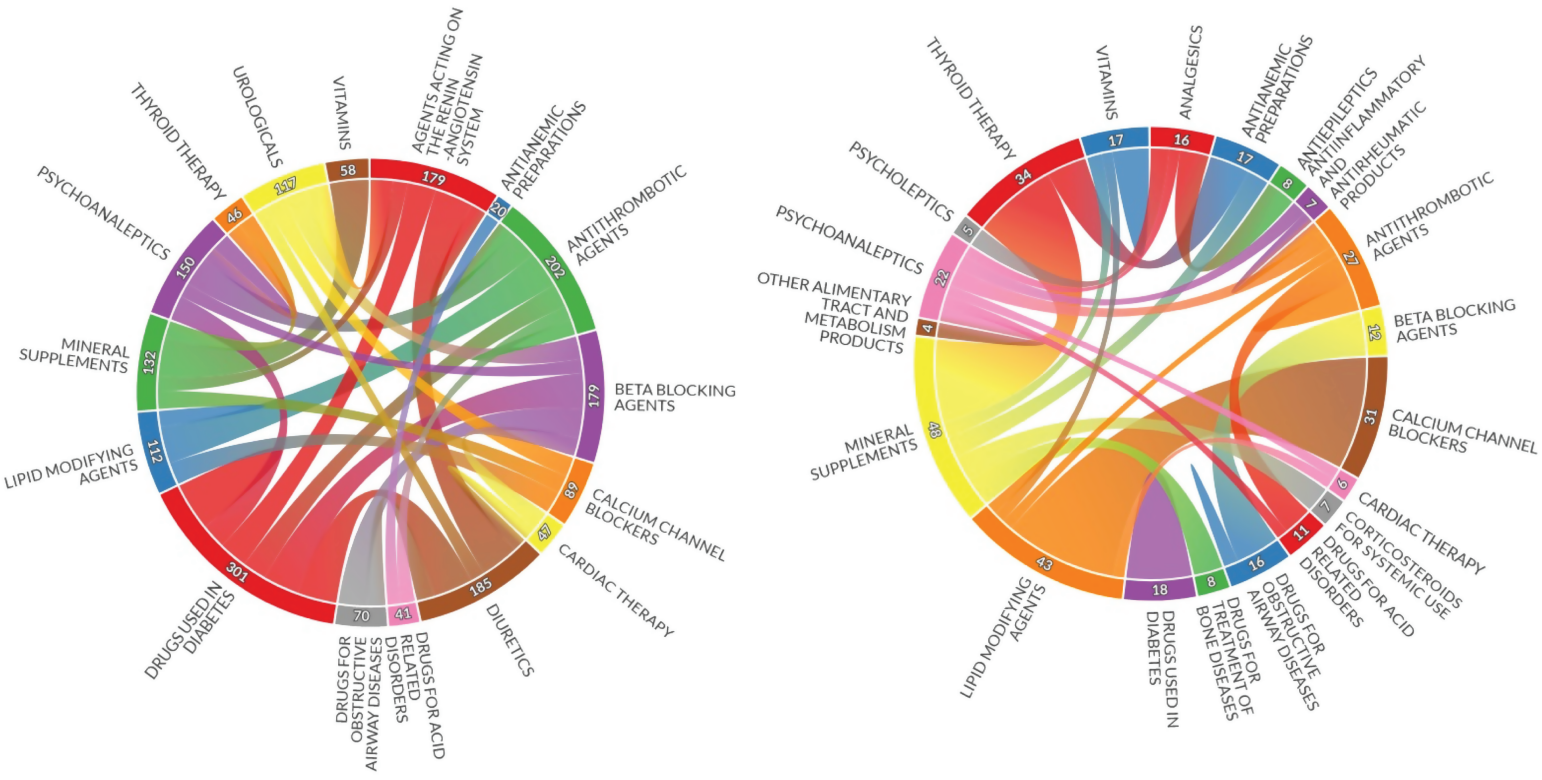
Chord diagrams showing most common therapeutic subgroups (WHO ATC level 2 classification) involved in all potential drug-drug interactions (left) and potential major drug-drug interactions (right). Subgroup interactions with <20 (left) and <3 (right) occurrences are not shown.

The most common therapeutic subgroups involved in potential DDI were drugs used in diabetes (*n* = 301 occurrences), antithrombotic agents (*n* = 202), and diuretics (*n* = 185, Fig. 3 left). The most common subgroups involved in potential major DDI were mineral supplements (*n* = 48 occurrences), lipid modifying agents (*n* = 43), and thyroid therapy (*n* = 34, Fig. 3 right). At the individual medication level, the most common agents involved in potential DDI were lisinopril (*n* = 92 occurrences), furosemide (*n* = 77), and calcium (*n* = 77, Supplementary Fig. 1).

For the medication network analysis using therapeutic subgroup level for all potential DDI, there were 40 nodes, density was 0.28 (ie, of all possible connections between subgroups, 28% signified potential DDI), diameter was 3 (ie, it took no more than 3 steps to traverse from one subgroup to any other using DDI connections), and triadic closure was 0.47 (ie, if 2 subgroups were connected to a third via a potential DDI, the 2 subgroups were also connected approximately 50% of the time). The individual medication level showed multiple disconnected sub-networks with low density and large diameters, as expected for the total number of distinct medications in the dataset (*n* = 517). No prior similar analyses exist for comparison across networks.

### Association of Baseline Characteristics and Polypharmacy/PIM

**Figure 4.**
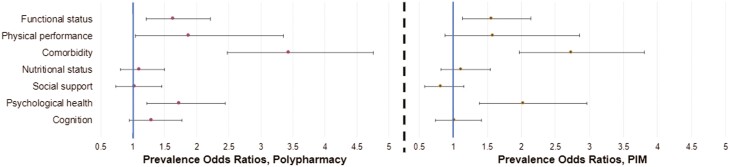
Association between impairment on geriatric assessment domains and: polypharmacy (≥5 meds, left) and PIMs (right). PIM, potentially inappropriate medications.

Patients with polypharmacy (≥5 medications) were more likely to be older (mean age 77.5 vs. 76.7), have a functional impairment (62.1% vs. 50.0%), be physically impaired (94.8% vs. 90.1%), have significant comorbidity (78.0% vs. 50.7%), and have impaired psychological status (32.7% vs. 21.9%). Table 1 and Fig. 4 (left) show prevalence odds ratios (PORs) for polypharmacy by baseline characteristics. Patients who received ≥1 PIM were more likely to be younger (mean age 76.7 vs. 77.8), have physician-estimated life expectancy ≤1 year (36.9% vs. 26.5%), have a functional impairment (61.0% vs.50.0%), have significant comorbidity (74.9% vs. 52.1%); and have impaired psychological status (33.0% vs. 19.5%). Table 2 and Fig. 4 (right) show PORs for PIMs by baseline characteristics.

**Table 2.** Association of baseline variables and PIM (≥1 high risk medication per Beers or STOPP criteria).

Variable	Category	All patients	PIM	No PIM	POR	95% CI	
*N*		718 (100%)	482 (61.3%)	236 (38.7%)			
Age, years	70-74	271 (37.7%)	190 (39.4%)	81 (34.3%)	Ref	—	—
	75-79	225 (31.3%)	149 (30.9%)	76 (32.2%)	0.84	0.57	1.22
	≥80	222 (30.9%)	143 (29.7%)	79 (33.5%)	0.77	0.53	1.13
Gender^a^	Male	405 (56.4%)	281 (58.5%)	124 (52.5%)	Ref	—	—
	Female	311 (43.3%)	183 (41.1%)	128 (47.2%)	0.78	0.57	1.07
Race^b^	White	628 (87.5%)	427 (89.1%)	201 (85.1%)	Ref	—	—
	Black	52 (7.2%)	29 (6.1%)	23 (9.8%)	0.59	0.34	1.05
	Others	35 (4.9%)	23 (4.8%)	12 (5.1%)	0.90	0.44	1.85
Education^a^	<High school	111 (15.5%)	81 (16.9%)	30 (12.7%)	Ref	—	—
	High school	244 (34.0%)	169 (35.2%)	75 (31.8%)	0.84	0.51	1.38
	College or above	361 (50.3%)	230 (47.9%)	131 (55.5%)	0.65	0.41	1.04
Income^a^	≤$50 000	371 (51.7%)	259 (54.0%)	112 (47.7%)	Ref	—	—
	>50 000	190 (26.5%)	119 (24.8%)	71 (30.1%)	0.73	0.50	1.05
	Declined to answer	155 (21.6%)	102 (21.3%)	53 (22.5%)	0.83	0.56	1.24
Marital status^a^	Single	17 (2.4%)	9 (1.9%)	8 (3.4%)	Ref	—	—
	Married/domestic partnership	449 (62.5%)	312 (65.0%)	137 (58.1%)	2.02	0.77	5.36
	Separated/widowed/divorced	250 (34.8%)	159 (33.1%)	91 (38.6%)	1.55	0.58	4.17
Cancer type	Gastrointestinal	246 (34.2%)	162 (33.9%)	84 (35.6%)	Ref	—	—
	Genitourinary	109 (15.2%)	65 (13.5%)	44 (18.6%)	0.74	0.41	1.34
	Gynecological	43 (6.0%)	28 (5.8%)	15 (6.4%)	0.76	0.48	1.21
	Breast	56 (7.8%)	33 (6.9%)	23 (9.7%)	0.96	0.49	1.90
	Lung	180 (25.1%)	135 (28.0%)	45 (19.1%)	1.55	1.01	2.37
	Lymphoma	46 (6.4%)	34 (7.1%)	12 (5.1%)	1.46	0.72	2.97
	Others	38 (5.3%)	21 (4.5%)	17 (6.3%)	0.95	0.46	1.96
KPS	20-60	93 (13.0%)	67 (13.9%)	26 (11.1%)	Ref	—	—
	70-80	379 (52.8%)	271 (56.3%)	108 (46.0%)	**0.97**	**0.59**	**1.61**
	90-100	244 (33.9%)	143 (29.7%)	101 (43.0%)	**0.55**	**0.33**	**0.92**
Life expectancy^c^	≤12 months	238 (33.1%)	176 (36.9%)	62 (26.5%)	Ref	—	—
	>12 months	473 (66.0%)	301 (63.1%)	172 (73.5%)	**0.62**	**0.44**	**0.87**

Two patients had missing data.

Three had missing data.

Seven had missing data.

Abbreviations: CI, confidence interval; KPS, Karnofsky Performance Status; PIM, potentially inappropriate medications; POR, prevalence odds ratio; STOPP, Screening Tool of Older Person’s Prescriptions.

Bolded values are statistically significant.

### Association of Polypharmacy/PIM Variables and Potential Drug-Drug Interaction

Polypharmacy, excessive polypharmacy, and PIM were all associated with significantly increased odds of potential major DDI. There was no significant association between polypharmacy (≥5 vs. <5 medications) or PIM (≥1 vs. none) and the odds of potential major DCI (Table 3). However, examining medication usage as a continuous variable, each additional medication (prescription and nonprescription) increased odds of a potential major DDI and DCI by 39% (*P* < .01) and 12% (*P* < .01), respectively. Each additional prescription medication increased these odds by 40% (*P* < .01) and 19% (*P* < .01), respectively.

**Table 3.** Association of polypharmacy/PIM variables and potential drug-drug and drug-cancer treatment interactions.

Variable	Definition	Any potential drug-drug interactions		Any potential major drug-drug interaction		Any potential major drug-cancer treatment interaction	
		POR	95% CI	POR	95% CI	POR	95% CI
Polypharmacy^a^	< 5 meds	Ref.	—	—	—	—	—
	≥ 5 meds	**19.58**	**12.78-28.71**	**5.64**	**3.55-8.95**	1.89	0.91-3.94
Polypharmacy (prescription only)	<5 meds	Ref.	—	—	—	—	—
	≥5 meds	**17.11**	**10.11-28.94**	**3.21**	**2.25-4.57**	1.49	0.78-2.85
Excessive Polypharmacy^a^	< 10 meds	Ref.	—	—	—	—	—
	≥ 10 meds	**29.71**	**7.26-121.56**	**6.74**	**4.32-10.49**	1.84	0.85-4.00
PIM^a^	No	Ref.	—	—	—	—	—
	Yes	**3.19**	**2.30-4.42**	**1.58**	**1.10-2.27**	1.23	0.62-2.43

Includes both prescription and nonprescription medications.

Abbreviations: CI, confidence interval; PIM, potentially inappropriate medications; POR, prevalence odds ratio.

Bolded values are statistically significant.

## Discussion

This study details the medication usage of 718 vulnerable older adults with advanced cancer starting a cancer treatment regimen with a high risk of toxicity in the community oncology setting. Over 61% of patients had polypharmacy (≥5 medications), and nearly 15% had excessive polypharmacy (≥10 medications). In other studies of older adults with cancer, prevalence of polypharmacy ranges from 2% to 80% depending upon the specific population studied and the definition of polypharmacy.^29^ Our study also reveals 67.1% of patients taking ≥1 PIM by either Beers or STOPP criteria, which is higher than other available studies where estimates range from 19% to 52% (based only on Beers criteria in most prior studies).^29^ Polypharmacy and PIMs are markedly prevalent in this cohort, which is more likely than prior studies to be representative of older adults with advanced cancer in the community setting (where most older adults are treated).

Unlike prior studies in older adults with cancer, this study reports detailed descriptive data regarding the types of medications these patients report taking regularly. Cardiovascular medications comprised nearly half of the prescribed medications reported. Interestingly, more than a quarter of the medications that patients reported taking regularly were non-prescription medications; most previous studies have only counted prescription medications, another likely reason for underestimation of PIMs. Non-prescription medications accounted for approximately 40% of PIMs detected, including common medications such as PPIs, NSAIDs, and antihistamines. Older adults may incorrectly assume that OTC medications are safe for them, and providers may be unaware of the full complement of medications their older patients are taking if a prescription was not generated. This study, therefore, helps delineate the size and shape of a problem underrecognized by both providers and patients,^30^ and highlights an opportunity for improved medication reconciliation, patient and caregiver education, deprescribing, and other interventions.^31^

Patients with polypharmacy and PIMs were more likely to have a higher comorbidity burden, functional impairment (as measured by KPS and GA), and impaired psychological status (including anxiety and depression). These associations coincide with those seen in prior studies in older adults with cancer.^2,18,29,32^ Although this study does not provide evidence of causality, it suggests opportunities for further prospective work (for example, determining the effect of interventions for polypharmacy/PIM, like deprescribing, on physical and psychological functioning). Although patients with polypharmacy in this cohort were older on average, patients taking ≥1 PIM were younger on average and more likely to have a life expectancy of less than 1 year. A landmark study demonstrated the safety of statin discontinuation in older adults with cancer and limited life expectancy,^33^ and the OncPal deprescribing guideline was developed to assist clinicians in identifying chronic medications that may be reasonable to deprescribe in older adults with advanced cancer.^34^ Discussions around goals of care in these patients should also include conversations about medication goals. Most older patients are willing to discontinue medications after discussion with their physicians,^35^ and may derive physical and financial benefit from doing so.

This study also highlighted the prevalence of potential DDI and DCI in this population. Almost 70% of patients in this cohort were at risk of DDI, and about one quarter was exposed to a potential “major” DDI, indicating that risks may outweigh the benefits of the medication combination. Nearly 5% of patients were taking medication combinations that are contraindicated, and a similar number were taking a medication that could interact with their chemotherapy regimen. These results are similar to the percentages seen in both the ELCAPA cohort of 442 patients ≥70 years starting antineoplastic therapy in France,^36^ as well as similar cohorts in the United States (*n* = 244)^16^ and Korea (*n* = 301).^37^ Considering medications interacting as analogous to a social network, the utilization of network analysis and graph theory offers a unique understanding of these data; to our knowledge, this method has not been previously reported to understand medication usage in older adults with cancer, and benchmarks do not yet exist for these types of networks. This analysis reveals that, although the network has sparse connections at the medication level (ie, only approximately 1% of the possible combinations have potential interactions), patients taking drugs from multiple therapeutic subgroups have a high risk of DDI (as 28% of therapeutic subgroup combinations result in DDI, and a high triadic closure suggests a high risk of multiple DDI within a single patient taking medications from multiple therapeutic subgroups). Older adults are more susceptible to adverse drug events (ADEs) compared to younger adults, due to polypharmacy, changes in organ function and drug metabolism, and other physiologic changes of aging. Approximately 10% of hospital admissions for older adults are associated with ADEs,^38,39^ with most of the hospitalizations considered preventable.^40^ In older patients with cancer receiving chemotherapy, polypharmacy is associated with dramatic increases (up to 114%) in unplanned hospitalizations.^41,42^ In the ELCAPA cohort, potential DDI (but not polypharmacy) was independently associated with the risk of unplanned hospitalization, suggesting that much of the risk may be attributable to DDI and suggesting an opportunity for further study and intervention. In our study, the risk of potential major DDI increased 39% with each additional medication (prescription or nonprescription), and the risk of an interaction with cancer treatment increased 12%, indicating a need to critically evaluate the utility and safety of every medication at the start of cancer treatment.

This study has a significant limitation in that it is a secondary analysis of a randomized clinical trial that was not designed to specifically study medication usage. However, extensive information about medications was captured with high fidelity and validity, including data about nonprescription medications (which are often unreported). Compared to other studies, which are often conducted in academic centers with fit older patients, this cohort of older adults with at least one impairment other than polypharmacy may provide more representative data. This study also provides an in-depth analysis of the medications most likely to be PIMs and/or to cause DDI/DCI in this cohort, suggesting targets for further intervention and study. More work is urgently needed to implement and evaluate interventions addressing polypharmacy and PIMs in older adults with cancer, particularly those initiating cancer treatment.^43^

## Supplementary Material

oyac053_suppl_Supplementary_Figure

oyac053_suppl_Supplementary_Table

## Data Availability

The data underlying this article will be shared on reasonable request to the corresponding author.
