# Supplementary material for: Polypharmacy, Potentially Inappropriate Medications, and Drug-Drug Interactions in Vulnerable Older Adults With Advanced Cancer Initiating Cancer Treatment
Source: Oncologist. 2022 Mar 28;27(7):e580–8. doi: 10.1093/oncolo/oyac053 (PMC9255971; doi:10.1093/oncolo/oyac053)

**Supplemental Figure 1. Chord diagram showing individual medications involved in drug-drug interactions (DDI). DDI with <5 occurrences in this cohort are not shown.**


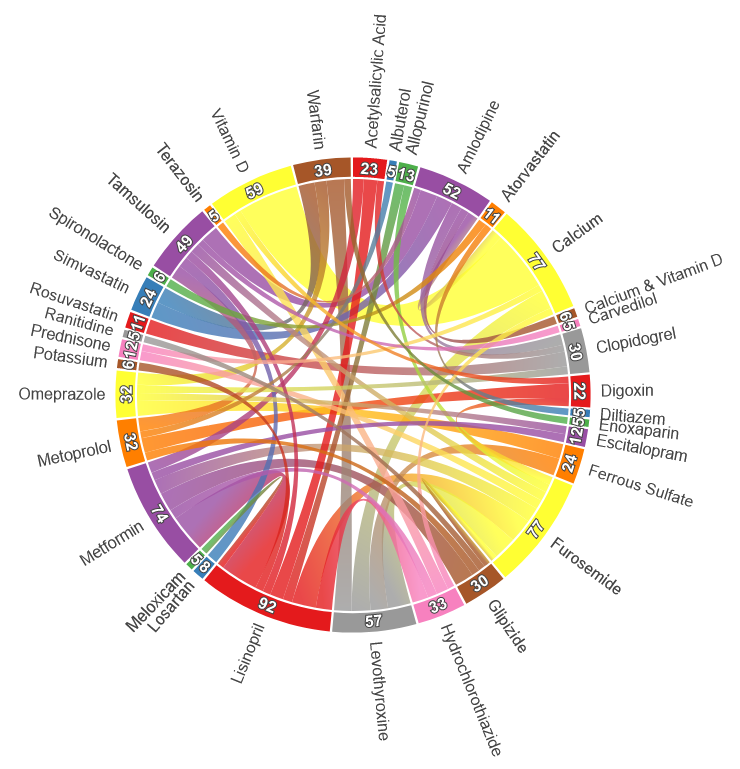

Supplement: oyac053_suppl_Supplementary_Figure [file oyac053_suppl_supplementary_figure.docx]
