# Supplementary material for: Polypharmacy, Potentially Inappropriate Medications, and Drug-Drug Interactions in Vulnerable Older Adults With Advanced Cancer Initiating Cancer Treatment
Source: Oncologist. 2022 Mar 28;27(7):e580–8. doi: 10.1093/oncolo/oyac053 (PMC9255971; doi:10.1093/oncolo/oyac053)
Supplement: oyac053_suppl_Supplementary_Table [file oyac053_suppl_supplementary_table.docx]

**Supplemental Table 1: Geriatric assessment domains, tools, and definitions of impairment**

| **Domains** | **Tools** | **Descriptions** | **Definition of impairment*** |
| --- | --- | --- | --- |
| ***Physical performance*** | *Timed "Up and Go” (TUG)* | Assess mobility over 3; longer time indicates worse performance | >13.5 seconds |
|  | *Short Physical Performance Battery (SPPB)* | Assess balance, gait speed, and strength; higher score indicates better performance (range 0-12 points) | ≤ 9 points |
|  | *Falls history* | Assess the number of falls | Any history of falls in the prior 6 months |
|  | *OARS Physical Health* | Assess any limitation in 12 activities (e.g. climbing several flight of stairs, walking more than a mile) as a result of his/her health (options: a lot, a little, not at all) | Patient answered “a lot” to any question |
| ***Functional status*** | *Activities of Daily Living (ADL)* | Assess difficulty with the following 6 activities: bathing, dressing, eating, getting in and out of bed/chairs, walking, toileting (options: yes/no) | Any deficit (answers “yes” to any question) |
|  | *Instrumental ADLs (IADL)* | Assess independence in the following 7 activities: using the telephone, transportation, shopping, preparing meals, doing housework, taking medicine, managing money (options: without help, with some help, completely unable to do) | Any deficit (answers any question as “with some help” or “completely unable to do”) |
| ***Comorbidity*** | *OARS Comorbidity* | Assess the presence of 13 illnesses (e.g. other cancer or leukemia, arthritis, glaucoma) as well as hearing and visual impairments, and how much each problem interferes with his/her activities (options: not at all, somewhat, a great deal) | Patient answered "yes" to 3 illnesses OR answered that 1 illness interferes "a great deal" (including eyesight and |
| ***Cognition*** | *BLESSED*  *Orientation- Memory- Concentration (BOMC)* | Assess orientation, memory, and concentration using 6 items and scores are weighted; higher score indicates worse performance (range 0-28 points) | ≥ 11 points |
|  | *MiniCog* | Assess word recall and clock drawing based on 3 items; lower score indicates worse performance (range 0-5 points) | 0 words recalled OR 1-2 recalled words + abnormal clock drawing test (or <3 points) |
| ***Nutritional status*** | *Body Mass Index (BMI)* | Divide weight in kilograms by height in squared | < 21 kg/m |
|  | *Weight loss* | Assess change in weight over 6 months | > 10% change in weight from 6 months ago |
|  | *Mini Nutritional Assessment (MNA)* | Assess nutritional status using 6 items; lower score is worse (range 0-14 points) | ≤ 11 points |
| ***Social Support*** | *OARS Medical Social Support* | Assess the presence of social support using 4 items (“someone to help if you were confined to bed, someone to take you to the doctor if needed, and someone to prepare your meals if you were unable to do it yourself, someone to help you with daily chores if you were sick.” Options: none of the time, a little of the time, some of the time, most of the time, all of the time) | Patient answers any one of the questions as "some of the time”, “a little of time”, or “none of the time" |
| ***Psycho-logical health*** | *Geriatric Depression Scale (GDS)* | Assess depression using 15 items; higher score is worse (range 0-15 points) | ≥ 5 points |
|  | *Generalized Anxiety Disorder-7 (GAD-7)* | Assess anxiety using 7 items; higher score is worse (range 0-21 points) | ≥ 10 points |

***Impairment is considered present within each domain if there is one impairment noted on at least one tool**
